# Regeneration and reprogramming compared

**DOI:** 10.1186/1741-7007-8-5

**Published:** 2010-01-20

**Authors:** Bea Christen, Vanesa Robles, Marina Raya, Ida Paramonov, Juan Carlos Izpisúa Belmonte

**Affiliations:** 1 Center for Regenerative Medicine of Barcelona, 08003 Barcelona, Spain; 2 Gene Expression Laboratory, The Salk Institute for Biological Studies, La Jolla, California 92037, USA; 3 Current address: INDEGSAL, University of León, 24071 León, Spain; 4 Current address: Department of Molecular Biology, University of León, 24071, León, Spain

## Abstract

**Background:**

Dedifferentiation occurs naturally in mature cell types during epimorphic regeneration in fish and some amphibians. Dedifferentiation also occurs in the induction of pluripotent stem cells when a set of transcription factors (*Oct4, Sox2, Klf4 *and *c-Myc*) is over expressed in mature cell types.

**Results:**

We hypothesised that there are parallels between dedifferentiation or reprogramming of somatic cells to induced pluripotent stem cells and the natural process of dedifferentiation during epimorphic regeneration. We analysed expression levels of the most commonly used pluripotency associated factors in regenerating and non-regenerating tissue and compared them with levels in a pluripotent reference cell. We found that some of the pluripotency associated factors (*oct4/pou5f1, sox2, c-myc, klf4, tert, sall4, zic3, dppa2/4 *and *fut1*, a homologue of *ssea1*) were expressed before and during regeneration and that at least two of these factors (*oct4, sox2*) were also required for normal fin regeneration in the zebrafish. However these factors were not upregulated during regeneration as would be expected if blastema cells acquired pluripotency.

**Conclusions:**

By comparing cells from the regeneration blastema with embryonic pluripotent reference cells we found that induced pluripotent stem and blastema cells do not share pluripotency. However, during blastema formation some of the key reprogramming factors are both expressed and are also required for regeneration to take place. We therefore propose a link between partially reprogrammed induced pluripotent stem cells and the half way state of blastema cells and suggest that a common mechanism might be regulating these two processes.

## Background

Differentiation during development is normally viewed as a one way process from undifferentiated to more differentiated cells. However, some lower vertebrates such as teleost fish and some amphibians are able to compensate for the loss of body parts by regenerating a nearly perfect copy of the original part by dedifferentiating cells *in vivo *to facilitate regeneration.

After the loss of an appendage undifferentiated, pluri- or multipotent cells from different origins accumulate at the damaged surface to form a regeneration blastema. The blastema is formed after wound closure through dedifferentiation of at least three terminally differentiated cell types, fibroblasts [1], keratinocytes [2] and myotubes [3]. Endogenous stem cells like muscle satellite cells also seem to provide cells for the blastema [4]. Despite the heterogeneous origin of the blastema cells, histologically they appear as a homogeneous population of cells and therefore have been traditionally viewed as a single cell type. This view however has been recently challenged [5]. After blastema formation, a period of extensive proliferation of blastema cells follows, before the cells re-differentiate to produce all the different cell types for the tissues of the missing appendage.

In contrast, it only recently became possible to dedifferentiate or reprogram somatic cells to pluripotent cells in vitro [6]. Exposure to just four transcription factors (most commonly *Oct4, Sox2, c-Myc *and *Klf4*) is enough to reprogram fibroblasts and many other differentiated cell types into induced pluripotent stem (iPS) cells [7-11]. This raises the question whether the *in vivo *dedifferentiation or reprogramming seen during regeneration has similarities to the *in vitro *reprogramming of fibroblasts to iPS cells.

We noticed that two of the reprogramming factors were expressed during *Xenopus *limb regeneration. Furthermore, a recent publication presented evidence that the reprogramming factors *c-myc, sox2 *and *klf4 *were expressed during regeneration in newts [12]. Therefore we thought to investigate the similarities and differences on a more systematic and broader scale.

In this paper we explore the possibility of similarities between reprogramming and regeneration from a molecular point of view. We chose two of the current regeneration models, zebrafish and *Xenopus *for this purpose. Each model offers different opportunities and techniques that add to the general picture of blastema cell formation. In particular we concentrated on caudal fin regeneration in zebrafish and limb and tail regeneration in *Xenopus*. While these three structures are very different anatomically it has been shown that the underlying molecular mechanism of regeneration is very similar [13-18], therefore conservation of the differentiation status of the blastema cells of these three appendages is as well expected. We focused on the studies of gene expression by quantitative real time polymerase chain reaction (qPCR) of regenerating and non-regenerating tissue compared to an embryonic, pluripotent reference cell type, analysis of the blastema cell cycle by fluorescence-activated cell sorting (FACS) and a functional approach by knocking down *pou5f1/oct4 *and *sox2 *with morpholinos in the zebrafish caudal fin demonstrating that some of the core factors needed for reprogramming are present and required during regeneration.

## Results

### Expression of pluripotency associated markers

To determine whether there are any similarities between *in vivo *regeneration and reprogramming of fibroblast cells to iPS cells we made two assumptions. First, we treated the blastema as a homogeneous population of cells, as would be predicted if they dedifferentiated to a pluripotent state and second, we expected overall levels of pluripotency associated factors to increase as abundance of blastema cells increases up to a comparable expression level of a pluripotent reference cell.

To assess the differentiation status of blastema cells a panel, consisting of zebrafish and *Xenopus *homologues of the genes most commonly used to characterize mouse or human ES and iPS cells, was put together. The panel includes the four most commonly used reprogramming factors *oct4 *(*pou class 5 homoeobox1*, also called *pou5f1*), *sox2 *(*sex determining region Y box 2*), *klf4 *(*kruppel-like factor 4*), *c-myc *(*proto-oncogene myc*) as well as *sall4 *(*sal-like4*), *lin28*, *α1, 3/4 fucosyltransferase lewis1 *(*Fut1*) which is the homologue of *stage-specific embryonic antigen-1 *(*ssea1*) and others (See Table 1). The panel, however, does not include *nanog*, a main player for pluripotency acquisition [19], since no homologue has been described either in zebrafish or *Xenopus*. For *oct4*, four homologues have been described in *Xenopus *(*oct25, oct60, oct79 *and *oct91*) and it has been shown for three of them that they can rescue the phenotype of an *oct4*^-/- ^knockout mouse embryonic stem (ES) cell line [20].

**Table 1:** Gene panel.

Gene	GenBank Acc no	function	Primer F	Primer R
**Zebrafish**				

pou5f1	NM_131112,1	pluripotency, homolog of mammalian Oct4	GGTTCGGAAGCCCAGGATT	TGAGCTGAGGGAATGTTTTGC

sox2	NM_213118,1	pluripotency	ACCCCGGAGGAAAACCAA	CCCGGCAGGGTGTACTTG

zic3	NM_001001950,2	pluripotency	CCCTGGGCTGGGACTCA	CTTGAAGGCAGCCGAGTGA

klf4	NM_131723	pluripotency	GAACCACTGCGGGCAAAT	GATGGTGGAGTCAGCATCACA

c-myc	L11710	pluripotency	CGTCAACGCGGCATGA	GATTGTTGCTAGCCTCAAGTCGTA

sall4	NM_001080609	Sall4 activates *Pou5f1 *expression *in vivo *and *in vitro*	CTCCCAGAGACCTTCTTCATCAG	GACCGAACATGCCAGAAGAAA

tert	NM_001083866	pluripotency, downregulation of *TERT *caused loss of pluripotency and human ESC differentiation	CGACAGCAAACCGAAAAAACTT	CGACTGAATAGCGGCACCAT

mps1 (or tkk)	NM_175042	blastemal proliferation	TGGATGGTTCGCTGAAGCTAA	GGTCACGTCAGGCTGAATCTG

hsp60	NM_181330	fin regeneration	GGTGAGGACGGCACTGCTA	TTCAGCGGTGGACAAGAGAGA

Msxb	NM_131260	regulate the rate of proliferation of blastema cells during fin regeneration	CCAGCAGGTCGCGTGTTC	TCCTGACCATGTCCCATTCTC

hsp90a1	NM_131328	mouse Es cells	TGAACTGATCCCAGACCAGAAA	CAATGCCGGTGTCGATGAT

**Xenopus**				

oct25	M60074	homolog of mammalian Oct4	CCCCAATGTTTCAGGCTTGT	CCACAGGCCGTGCAGACT

oct60	M60075	homolog of mammalian Oct4	CAGAAACACAGCCGGACAGA	CACCCATAGCAGCACAGCAT

oct79	M60076	homolog of mammalian Oct4	CACGACCTGACCTCCTGGTATAC	TGCTGGACCTCCATTAATATTGC

oct91	M60077	homolog of mammalian Oct4	AACGTGACCTCGATTTGCACTA	AACGTGACCTCGATTTGCACTA

sox2	NM_001088222	pluripotency	GCACATGTCGCAACACTATCAGA	GGCAGCGTGCCATTGATC

c-myc	X56870	pluripotency	GGCGGAACGAGCTTAAGTTG	CGCCACCTCGGGTACCT

lin28	NM_001087449	pluripotency	GCCCAGTGTCCAGAGAAAGC	TCCTCAGTGATTGGCTGATCTTC

zic3	NM_001087619	pluripotency	TGCCAGCTCAGGGTACGAAT	TCCTCACTGTTGGCAGAAACC

tert-A	NM_001085633	pluripotency, homolog of human htert	ATATTCTTGCTTCAAGCTTACAGGTTT	CCGCTGGCCAAATGGA

cripto3	AJ864901	pluripotency, homolog of TDGF3	GGAAGATATTGCGAGCTTCATGT	CAATGGCCATGTGGAACAATT

gdf3	NM_001087497	pluripotency	GCTGTTTTACAGACTCTGGTGCAT	GGGCACAGCAAGGCAATG

sall4	AY336983	pluripotency	AGTTCATTGTGCCTCCTACAGTCA	CGCGGTGGCATACTGGTT

dppa2/4	NM_001096099	pluripotency, hypothetical protein equally homolog to dppa2 and dppa4	TGTGACGAGTGTTTTGAGAGGAA	AGACCGACCTCTGCTGCAA

Fut1	AY278679	pluripotency, α1,3/4 fucosyl transferase lewis 1, mouse homolog of ssea1	TGCCACCTTACGAACTTTCTCA	AATCTGTCCCGTTTCACTTTGC

msx1	BC081101	blastema marker	GAGAAAGCACAAAACCAACAGAAA	GGCCAGCAGTTGGGATGTAG

fgf8	NM_001090435	blastema marker	CGACCCACACGCCAAGTTA	TTTAATGCGAACTCTGCTTCCA

lef1	NM_001096734	blastema marker	CCCACTGTCCCCAGGAAGT	GTGGATACCAGCCCAATGGT

ODC	NM_001086698.1	housekeeping gene	TCCATTGAGAGCGTAGGACTTG	GAGGCTCGCCGGTGAAATA

Forward and reverse primers used in quantification of relative expression by qPCR of zebrafish and *Xenopus *genes. Gene names and accession numbers are specified as well as gene function.

We first tested the panel of genes in a cell type representing the closest to a pluripotent cell type in these species. Since neither *Xenopus *nor zebrafish ES cell cultures have been properly established [21] we used cells from early developmental stages of both species.

For *Xenopu*s, animal cap cells were chosen as the pluripotent cell type, as these cells are able to differentiate into many different cell types of all three germ layers in culture by over-expressing specific genes or by culturing the caps in different conditions [22,23].

**Figure 1:**
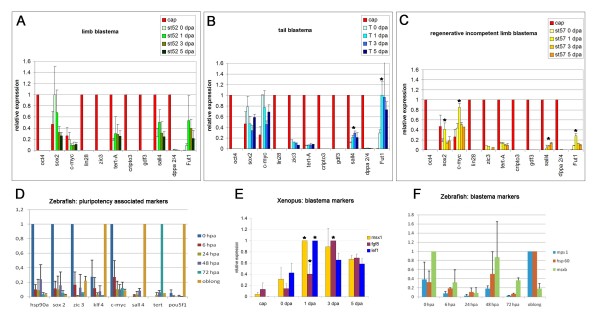
**qPCR of a pluripotent cell type compared with cells of the regeneration blastema**. **A) **Relative expression of pluripotency associated genes in *Xenopus *cap cells compared to limb blastema (st52) at different time points during regeneration. In red is relative expression of animal cap cells, in green the relative expression of st52 limb blastemas at 0 dpa, 1 dpa, 3 dpa and 5 dpa. (note: the results for *oct25, oct60 *and *oct79 *expression was combined as *oct4 *expression). **B) **Relative expression of pluripotency associated genes in *Xenopus *cap cells and st52 tail blastema cells. **C) **Relative expression of pluripotency associated genes in *Xenopus *cap cells and regenerative incompetent pseudo blastemas of st57 limbs at 0 dpa, 1 dpa, 3 dpa, and 5 dpa. Asterisks indicate significant (*P *< 0.05) upregulation of expression compared to 0 dpa. **D) **Relative gene expression for pluripotency associated markers in zebrafish non-regenerating (0 hpa), regenerating (6 hpa to 72 hpa) fin and embryonic cells in oblong stage. **E) **Relative expression of blastema markers in cap cells and regenerating limbs. Asterisks indicate significant (*P *< 0.05) upregulation of gene expression compared to the previous time point. **F) **Relative expression of blastema markers in zebrafish non-regenerating (0 hpa), regenerating (6 hpa to 72 hpa) fin and embryonic cells in oblong stage. For each gene, expression was normalized to highest expressing tissue. Error bars indicate Standard deviation (SD). (For statistical differences see Additional file 1).

All but two of the pluripotency associated markers tested in *Xenopus *were expressed in the animal cap cells indicating that a similar gene core network might be operational during early amphibian development to confer pluripotency (Figure 1A) to that found in the maintenance of pluripotency of mouse or human ES/iPS cells [24-27]. The exceptions were *Fut1*, the homologue of *ssea1*, and *oct91 *which did not show expression above the rt^- ^control. (The results for *oct25, oct60 *and *oct79 *were combined as *oct4 *in Figure 1A-1C since they all showed similar expression profiles while *oct91 *was left out of Figure 1A-1C)

To cover the complete process of *Xenopus *limb and tail regeneration we chose four discrete time points that represent different stages of regeneration to assess the differentiation status of blastema cells: non-regenerating tissue (0 day post amputation (dpa)), blastema formation (1 dpa), blastema expansion (3 dpa) and blastema redifferentiation (5 dpa). If cells acquired pluripotency during regeneration one could expect to see an increase in pluripotency associated markers either during blastema formation (1 dpa) or blastema expansion (3 dpa). Since, *Xenopus *loses the capacity to regenerate its limbs during metamorphosis (stage (st) 56 to 63) [28,29], we included regenerative incompetent limbs at st57 as a further control in our study.

The expression profile of the same factors in *Xenopu*s blastema cells showed some differences to animal cap cells. Out of 11 factors tested only six or seven were expressed in a limb or tail blastema, respectively and each of these factors was also expressed in both non-regenerating limbs (0 dpa) and regenerative incompetent limbs (st57) (Figure 1A-1C).

Of these seven genes, four (*zic3, tert-A, dppa2/4, sall4*) were expressed at lower levels in the blastema than in pluripotent cells, while *sox2 *and *c-myc*, two of the factors facilitating reprogramming, and *Fut-1*, an early marker in the reprogramming process, were expressed at similar or higher levels. Not expressed above rt^- ^control were the homologues of *oct4 *(*oct25, oct60, oct79, oct91*), *lin28, cripto3 *and *gdf3 *(Figure 1A and 1B).

Furthermore, none of the factors tested was upregulated during limb regeneration. However, some tissue specific differences between tail and limbs were found. Two of the factors, *sall4 *and *Fut1*, displayed modest upregulation during tail blastema formation, but no statistically significant upregulation during limb regeneration even though both factors showed a similar expression wave over time in tail and limb regeneration. (see Figure 1B and 1A). To our surprise we also found some upregulation of *sox2, c-myc *and *Fut1 *in regenerative incompetent pseudo blastemas of st57 limbs that peaked at 1 dpa and dropped back to expression levels of non-regenerating (0 dpa) limbs by 3 dpa. Since this peak of expression was not seen with all the genes tested, we assumed that it is biologically real and not caused by a technical error. Furthermore, sall4 is also significantly upregulated at 3 dpa and 5 dpa in the regenerative incompetent limbs (Figure 1C). We do not know what the significance of these expression peaks are, however they are certainly not relevant for pluripotency in these regenerative incompetent pseudo blastemas. A similar early peak of *sox2 *expression has also been found in the non-regenerating ventral iris during lens regeneration in newts [12].

To test the quality of our dataset, three other genes that have been described as blastema markers (*msx1, fgf8 *and *lef1*), were tested and showed the expected upregulation of expression during the first 5 dpa (Figure 1E) giving us confidence in the quality of the dataset [13,30-32].

Other studies have reported *sox2 *and *c-myc *expression in newt lens and limb regeneration [12]. While these authors report upregulation of the two factors in newt limbs, we did not see a significant change in expression levels during regeneration in *Xenopu*s. Moreover, the two factors were also expressed in regenerative incompetent limbs in *Xenopus*, with *c-myc *being expressed at much higher levels in the older limb than in the younger, regenerative competent limb.

For zebrafish, embryos at the oblong blastula stage (3 2/3 hpf at 28.5°C) were chosen to represent the pluripotent cell type since this stage is considered optimal to try to derive zebrafish ES cells [33]. These blastula derived cells have been shown to be able to contribute towards the germ line if injected into zebrafish embryos and therefore to be pluripotent [34].

In accordance, we found that all eight pluripotency related genes tested were expressed in these blastula cells (Figure 1D and Additional file 1), with three of them (*pou5f1, klf4, sall4*) expressed at significantly higher levels than in early regenerating fin cells. The other five genes (*sox2, c-myc, tert, zic3, hsp90a*) were expressed at low levels, both in the embryonic cells and in non-regenerating fin, with only *sox2 *showing significantly higher expression in fin than in embryonic cells (Figure 1D). We noticed, though, much higher *sox2 *expression in a slightly younger blastula stage (unpublished result) but not in oblong stage.

In agreement with results obtained in *Xenopus*, none of the eight pluripotency associated markers tested showed any upregulation during wound healing (6 h), blastema formation (24 h, 48 h) or blastema outgrowth (72 h). Intriguingly, *pou5f1 *was not completely shut down in adult fins and was also expressed in the blastema, while no expression was found in blastemas of either newts or *Xenopus *([12], this paper). Some of the fin blastema markers were unexpectedly highly expressed also at oblong stage (Figure 1F).

These results demonstrate that expression of pluripotency associated genes alone does not indicate pluripotency of that cell, since all of these genes are also expressed in differentiated cells of the limb, tail and fin at similar levels.

To make sure we did not miss any relevant gene in our panel, we investigated the relative expression of pluripotency associated genes in previously published microarray data sets from regenerating *Xenopus *limbs and cap cells and zebrafish caudal fin (see Methods). We found good agreement with our qPCR results in that the relative expression of pluripotency associated markers was generally higher in animal cap cells than in *Xenopus *blastema cells and in particular they were not upregulated upon amputation in limb blastema cells compared to non-regenerating limbs. One notable difference though is, that *oct91 *was found to be more highly expressed in animal cap cells, but similarly was not expressed or very lowly expressed in blastema cells. The difference might originate from a suboptimal primer set used in qPCR analysis.

The zebrafish caudal fin qPCR results too, were in agreement with published microarray data from regenerating adult fins. Generally the stem cell markers were expressed both in regenerating and non-regenerating fins and none was upregulated in blastema cells, while the relative expression of blastema markers was higher in regenerating adult fins [35]. This confirms that our qPCR results represent the general trend.

### Spatial expression pattern of pluripotency associated stem cell markers

For function, not only absolute expression level but also spatial distribution of a factor matters. Therefore, we investigated the spatial expression of some of the reprogramming and pluripotency associated factors in *Xenopus *looking at the expression patterns of the three factors that showed the highest absolute real time expression (*sox2, c-myc *and *sall4*).

**Figure 2:**
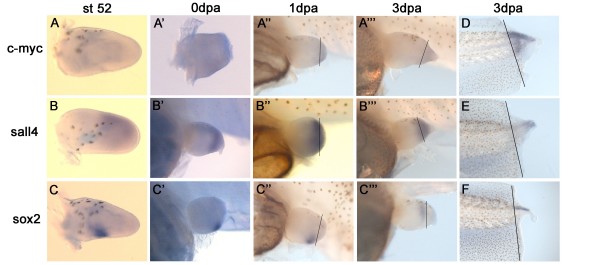
***In situ *hybridization of *c-myc, sall4 *and *sox2 *in developing and regenerating limbs and tails**. **A-A"') **Expression of *c-myc *in developing st52 limbs (A) and amputated limbs at 0 dpa, 1 dpa and 3 dpa (A'-A"'). c-myc expression is diffuse and very weak in the developing limb bud and the blastema. **B-B"') **Expression of *sall4 *in developing st52 (B) and regenerating limbs at 0 dpa, 1 dpa, 3 dpa (B'-B"'). *sall4 *is expressed diffusely in the distal mesenchyme of the developing limb bud and after amputation in the whole of the blastema. **C-C"') **Expression of *sox2 *in developing (C) and regenerating (C'-C"') limbs. *sox2 *is expressed proximal to the blastema in the posterior mesenchyme. **D) ***c-myc *mRNA is expressed in the entire regeneration bud of a 3 dpa tail and is much stronger than in limbs. **E) ***sall4 *mRNA expression in 3 dpa tail is mainly seen in the distal tip of the regenerating tail. **F) ***sox*2 mRNA expression in 3 dpa tail is confined to the neural tube. (dpa = days post amputation). Thin black line indicates amputation plane

*In situ *hybridization revealed that *c-myc *was most highly expressed in the non-regenerating *Xenopus *tail and the tail blastema where it spread over the whole of the regeneration bud, including notochord, neural tube and mesenchyme (Figure 2D). The expression in the developing (Figure 2A) and regenerating limb bud (Figure 2A'-2A"' was much lower and diffusely spread over the whole limb bud, which matched with the qPCR result where expression was four times lower in the limb bud than in 3 dpa tail blastema.

The expression pattern of *sall4 *in developing and regenerating limb buds has been published previously [36] and is included here because it is one of the most highly expressed pluripotency associated genes in regeneration blastemas.

We found *sall4 *was localised to the distal mesenchyme of the developing st52 limb bud (Figure 2B) and was also strongly expressed in the mesenchyme of the regenerating limb (Figure 2B'-2B"'), which was in agreement with the published expression pattern. In the regeneration bud of the tail *sall4 *was mainly localized to the distal tip (Figure 2E).

In contrast, *sox2 *showed a very distinct localization to a posterior region in the developing limb bud just proximal to the amputation plane (Figure 2C). In the regenerating limb *sox2 *seemed still confined to the same region in the distal stump and showed little or no expression in the blastema itself (Figure 2C'-2C"'). During collection of blastemas for qPCR this distal stump region was included since dedifferentiation is thought to happen in this distal zone.

During tail regeneration *sox2 *expression was confined to the neural tube (Figure 2F).

The diffuse expression pattern seen for *c-myc *and *sall4 *is compatible with a model where *c-myc *and *sall4 *induce or facilitate dedifferentiation of the stump tissue and keep the cells in a less differentiated state. However, the localised expression pattern seen for *sox2 *points more towards a role in the development of a specific tissue or cell type in the neural tube and limb bud.

### Cell cycle analysis in regenerating and non-regenerating fin cells versus embryonic cells

Embryonic stem cells differ markedly from other cells in their cell cycle profile and regulation. It has been shown that ES cells and other pluripotent cells have much shorter gap phases (G1 and G2) or lack them altogether. These particular cell cycle properties of ES cells have been connected with pluripotency maintenance [37]. To compare cell cycle profiles of pluripotent blastomere cells and fin blastema cells, we performed a FACS analysis of their cell cycle.

**Figure 3:**
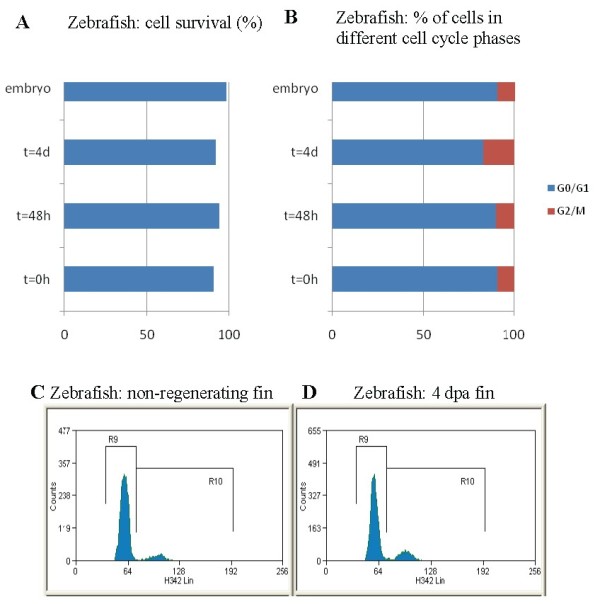
**Cell cycle analysis**. **(A) **Cell survival after blastomere and blastemal cell recovery at different time points (0 h, 48 h and 4 d). **(B) **Representation of cells in G0/G1 (blue) and G2/M (red) cell cycle phases. Results are expressed as percentages. FACS histogram of non-regenerating **(C) **and 4 dpa regenerating fin blastema cells **(D)**. Cells in G0/G1 are included in R9 and G2/M in area R10.

Cell survival after blastomere and blastemal cell recovery was high in all samples, ranging from 90% to 98% (Figure 3A). In all samples most of the cells were in G0/G1. The percentage of cells in G2/Mitosis (G2/M) was quite similar in the non-regenerating fin (t = 0 hours post amputation (hpa)), early regenerating fin (48 hpa blastema) and in the embryo (9.3%, 10.6% and 10.14% respectively) but higher in 4 dpa blastema cells (17.2%) where regenerative outgrowth is at its highest (Figure 3B, 3C and 3D).

While FACS profile of cells showed similar percentage of cells in G2/M at 0 hpa and 48 hpa, specific differences were observed in pH3 expression in localized zones when 0 hpa cells and 48 hpa blastemal cells were compared (Additional file 2) showing that cells in the blastema were more actively dividing than in the non-regenerating fin. On the other hand, histograms from flow cytometric analysis of Hoechst-stained cells did not show similarities between FACS cell profile in embryos and in the regenerating fin (data not shown) indicating that blastemal cells are controlled by a somatic cell cycle and do not revert to an embryonic one.

### Knockdown of either *pou5f1 *or *sox2 *impairs fin regeneration

Having shown the presence of the reprogramming factors, we wanted to test whether they were necessary for regeneration. To test for functionality we carried out a morpholino knockdown in the zebrafish caudal fin using two published morpholinos which had been tested in the embryo for specificity and function. We chose to use morpholino 1 (from now on called pou MO) against the *pou5f1 *gene [38]. Pou MO has been shown to phenocopy the mutant strain *spiel-ohne-grenzen *(*spg*), which has a mutation in the *pou5f1 *gene. The sox2 MO has been previously tested during retina pattern formation in zebrafish [39]. Since we did not know what phenotypes to expect for pou MO and sox2 MO injected fin blastemas, we used msxb morpholino, which has a published phenotype in fin regeneration as a positive control. The homeobox gene *msxb*, a frequently used blastema marker, has been shown to be indispensable for blastema growth with a morpholino knockdown method [40]. If we were able to replicate the published msxb MO phenotype in the fin then we could have confidence in the phenotype from the pou MO and sox2 MO.

**Figure 4:**
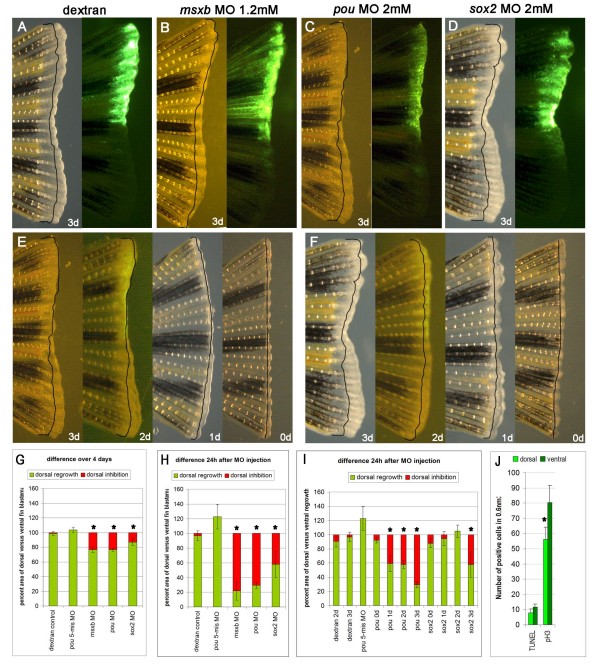
**Morpholino injection into zebrafish caudal fins**. **A-D) **Bright field and fluorescence picture of a dextran (A) msxb MO (B) pou MO (C) and sox2 MO (D) injected and electroporated fin: injections were done into the dorsal half of a 3 dpa blastema and photographed 24 h later. **E, F) **Time course for pou MO (E) and sox2 MO (F) injected and electroporated fins at 3 dpa, 2 dpa, 1 dpa and 0 dpa. Pictures were taken 24 h after injection. **G-I) **Percentage of dorsal versus ventral fin outgrowth (in green) and average inhibition of dorsal versus ventral fin (in red) of fins which were injected 3 dpa (G, H) and on day 0 to 3 pa as indicated (I). (see Method for exact calculation) (Asterisk indicate significant difference (*P *< 0.5) compared to dextran or pou 5-mis MO control or for 1 dpa injected fins between pou MO and sox2 MO injected fins). **J) **Measurement of apoptosis and cell proliferation in pou MO injected fins. There is no significant difference between dorsal and ventral fins in apoptosis as measured by TUNEL while there is a significant reduction of cell divisions in the dorsal fin as measured by pH3 staining (*P *≤ 0.05). Black lines indicate blastema size on day of injection. Error bars indicate standard error.

As a control for unspecific phenotypes induced by injection we injected the lineage tracer dextran and a pou MO with five mismatches (pou 5-mis MO). As expected neither dextran nor pou 5-mis MO had an effect on fin outgrowth (Figure 4A and 4G (The increase in dorsal regrowth seen with pou 5-mis MO is not significant)). Our positive control, msxb MO, injected into a 3 dpa blastema inhibited fin regeneration to a similar extent as previously published [40] (Figure 4B and 4G). When pou MO and sox2 MO were injected into the dorsal 3 dpa fin blastema an impairment of regeneration was observed in the dorsal fin (Figure 4C and 4D). While msxb MO reduced overall regrowth in the dorsal fin to 77% of ventral fin regrowth, pou MO and sox2 MO reduced dorsal regrowth over four days to 78% and 87%, respectively. However, if regrowth was calculated for the last 24 h only, when morpholino was actively present, the inhibition was much more pronounced, with dorsal regrowth down to 22%, 33% and 58% for msxb MO, pou MO and sox2 MO, respectively. This demonstrates that both *pou5f1 *and *sox2 *are not only present in the fin blastema but also necessary for regeneration at 3 dpa (Figure 4H).

Injection of msxb MO only showed a significant reduction of fin outgrowth if injected into a 3 dpa blastema in accordance with the observed increase in *msxb *expression around that time as detected by qPCR or *in situ *hybridization [41]. Reasoning that the reprogramming factors *pou5f1 *and *sox2 *might be already needed at an earlier time point during fin regeneration we injected morpholino at different times during blastema formation and regrowth. Injection of morpholino into 0 dpa fins had no effect on regeneration, though for technical reasons we were only able to inject much lower volumes than into blastemas themselves. Injection of sox2 MO into a 1 dpa regenerating fin also did not lead to a phenotype (Figure 4F and 4I). However, pou MO injected into 1 dpa blastema inhibited dorsal fin outgrowth by 40%. If morpholinos were injected into 2 dpa fin blastemas again pou MO but not sox2 MO reduced fin outgrowth by a similar extent as at 1 dpa (Figure 4E, 4F and 4I).

So while *msxb *and *sox2 *are only needed for fin regrowth at three days post amputation, *pou5f1 *has an additional function during the formation and outgrowth of the early blastema.

To see if the reduction in regrowth was due to cell death we performed terminal deoxynucleotidyl transferase-mediated dUTP nick end labeling (TUNEL) staining 24 h after injecting pou MO into 2 dpa fin blastemas to visualise dying cells. A count of defined areas of four different fins revealed no difference in numbers of dying cells between the dorsal and the ventral fin (Figure 4J). In contrast, when we analysed cell proliferation by pH3 immunostaining in 4 dpa fin blastemas injected with pou MO 24 h earlier, we could see a clear and significant reduction (*P *≤ 0.05) in cell divisions in the dorsal fin in three out of four fins (Figure 4J). The difference between dorsal and ventral cell divisions is not as big as expected because pH3 immunostaining only marks actively dividing cells at one particular time point. Furthermore dorsal outgrowth seems to recover over time since dorsal and ventral regrowth differences become less pronounced 48 h after MO injection.

## Discussion

### Restricted dedifferentiation of blastema cells

Appendage regeneration is a complex biological process that takes place only in a few lower vertebrates like teleost fish and some amphibians. A key step in the replacement of the lost appendage is the formation of the blastema, a transient structure of undifferentiated and proliferating cells which gives rise to all the different cell types in the newly formed appendage. However, how exactly blastema formation is achieved is still unknown. The idea that the process of dedifferentiation of fibroblast cells to iPS cells and dedifferentiation during *in vivo *regeneration is regulated by a similar mechanism is attractive but unexplored.

For that reason, expression profiles of pluripotency associated factors in blastemal cells were compared with expression profiles in pluripotent embryonic reference cells. A hallmark of vertebrate epimorphic regeneration is the re-activation of genes that are active during embryonic development [42]. We assumed that if blastema cells became pluripotent they would reactivate or upregulate the genes which are part of the pluripotency network, as is seen for iPS cells [25-27].

However, we found that blastemal cells had little in common with our pluripotent reference cells. Blastema cells neither upregulated any of the key transcription factors needed for induction of iPS cells (*pou5f1, sox2, c-myc, klf4*) as would be expected if they became pluripotent nor did they significantly upregulate many of the other transcription factors needed for self-renewal in iPS cells. Furthermore, hierarchical clustering of the published global gene expression data for *Xenopus *animal cap cells and blastema cells [43-45] demonstrated that they exhibit a different expression pattern, that is, blastema cells clustered on a different tree branch than pluripotent cells (data not shown). In other aspects as well, blastema cells were distinct from pluripotent cells. The analyses of the cell cycle showed that blastema cells have all the characteristics of somatic cells and cycle more slowly than pluripotent stem cells.

Furthermore, evaluation of the expression pattern of the three most highly expressed genes during *Xenopus *regeneration in the qPCR analysis revealed that one is not expressed uniformly in the blastema as would be expected if it had a role in conferring or maintaining pluripotency in these cells. This evidence suggests that blastema cells are not pluripotent but at best only multipotent. A similar conclusion was reached for newt blastema cells since neither *oct4 *nor *nanog*, two of the central factors in the network conferring pluripotency to iPS cells, were expressed [12]. Additionally, a recently published paper implies that blastema cells have less differentiation potential than thought [5]. Kragl et al. (2009) reported that the regeneration blastema in Axolotl is a pool of heterogeneous progenitor cells with restricted developmental potential, despite the rather uniform and undifferentiated appearance of blastema cells. By labelling specific tissues with embryonic tissue grafts marked with green fluorescent protein they could show that each tissue produces its own progenitor cells. These progenitors do not cross tissue boundaries during regeneration but only regenerate its own tissue type, so muscle regenerates muscle and cartilage regenerates cartilage, with the only exception of dermis which is able to make cartilage and dermis but not muscle. In accordance, a recent proteomics analysis of regenerating *Xenopus *limb buds revealed a significant number of upregulated proteins in blastema cells are also found in one or more adult stem cell types [46]. Whether this means that mature cell types dedifferentiate to become more like adult stem cells or whether resident adult stem cells are activated to produce blastema cells with hallmarks of the cells of origin is unclear at the moment.

### Possible mechanism and function

Despite the evidence that blastema cells are not pluripotent there are some strong similarities between blastema cells and iPS cells. Blastema cells and their precursors express low levels of some or all of the key factors needed for reprogramming to iPS cells. We hypothesise that these low levels are sufficient to trigger a similar mechanism to reprogramming which then gets stalled in an early, partially reprogrammed state leading to a cell which is only multipotent. Such intermediate states are frequently found during reprogramming among iPS colonies. Some inroads have been made in deciphering the mechanism of reprogramming by analysing this/these intermediate state/s (see review: [47]).

The process of reprogramming is slow and takes one to two weeks. Some of the earliest markers that are upregulated during this process are alkaline phosphatase (AP) and SSEA1. The SSEA1 expressing cells then activate other pluripotency associated genes like *Oct4, Sox2, Nanog *and *Tert *only late in the process. The two early markers (AP and *Fut1*, homologue of *ssea1*) are also either expressed or upregulated during blastema formation in *Xenopus *(not shown and Figure 1). Therefore it is plausible that these two genes might function in a similar way during dedifferentiation and blastema formation as during reprogramming by facilitating the action of the later factors.

In zebrafish, none of the pluripotency associated markers tested are completely shut off in the adult fin. Similarly in *Xenopus *seven of the 11 genes looked at are already expressed in the developing limb bud or tail. Avoiding a complete shutdown of gene expression should facilitate reactivation or reemployment of these factors later on, for example, during regeneration. Here we showed that these factors are indeed not just present but also required for regeneration. A knockdown of *pou5f1 *with a gene specific morpholino impaired fin regrowth at various time points during regeneration. And likewise a second gene, *sox2 *was also necessary for regeneration but to a lesser extent. From these results we conclude that, despite *pou5f1 *expression in zebrafish regenerating fin not reaching levels of a pluripotent cell, this gene is crucial for fin regeneration from the very early stages of regeneration onwards. At this stage, however, we can only speculate what that function is, either during regeneration or in the adult fin. It has been shown that the function of Pou5 proteins is conserved between mouse and *Xenopus *and that they regulate similar genes in ES cells and early *Xenopus *embryos [20]. The authors concluded that the ability of Oct4 to maintain ES cell pluripotency is derived from the ancestral function of this class of proteins to maintain multipotency during early vertebrate development. It is reasonable to assume that the low level of *pou5f1 *expression during regeneration is not enough to confer real pluripotency reverting the blastema cells to a completely reprogrammed state, however, that a synergistic effect of *pou5f1 *together with other pluripotency genes, could enable these cells to dedifferentiate and provide them with the multipotency required to give rise to certain cell types, allowing the reconstitution of the lost structure. However, while *pou5f1/oct4 *is required for fin regeneration, it does not seem to be essential for blastema formation in general, since neither *Xenopus *nor newt reactivate *pou5f1/oct4 *homologue expression in the blastema [12]. But then again reprogramming of neural stem cells has also been achieved without *Oct4 *as one of the reprogramming factors indicating that reprogramming can be successful without exogenous *Oct4 *[48].

In contrast to what was observed for *pou5f1, sox2 *seems to be mainly required for regenerative outgrowth similarly to what has been observed for *msxb*. Taking the expression pattern of *sox2 *in limb and tail blastema and its role in fin outgrowth into account we speculate that *sox2 *could have a role in specifying early neural precursor cells that contribute to the innervation of the regenerate. A role for *sox2 *in neurogenesis has been previously described in other species [49,50] and in mouse ES cells [51]. Furthermore, in a previous *in vitro *study we observed that *sox2 *is expressed in blastula cell colonies that will subsequently give rise to neuronal precursors (unpublished results). Additionally, limb and fin blastema outgrowth requires innervation to proliferate and replace structures [52-55].

Research into the mechanism behind reprogramming also demonstrated that the presence of the reprogramming factors alone was not sufficient for success since secondary iPS cells harbouring dox-inducible transgenes were only induced at a rate of 2% [56,57]. There are several lines of evidence that a correct stoichiometric mixture of the reprogramming factors is necessary for successful reprogramming and that more is not necessarily better. Leaving some of the factors out of the reprogramming cocktail can increase iPS efficiency in cells with expression of these factors [58]. Others have found that in an all-in-one vector approach, the sequence of the factors is important, again pointing towards the importance of delivering the correct relative level for each reprogramming factor. This could explain why low levels of the pluripotency associated factors might be sufficient to start the dedifferentiation/reprogramming process during blastema formation if the stoichiometric requirement is fulfilled. Furthermore, it could also account for why older, non-regenerating *Xenopus *limbs will not initiate blastema formation despite expressing the same factors but at different and ineffective stoichiometric combination.

## Conclusions

Knocking down the expression of *pou5f1/oct4 *and *sox2 *with morpholinos resulted in an impairment of regeneration, indicating that, despite the low expression levels, these factors do have a functional role during epimorphic regeneration. In the light of this functional evidence we make an associative link between partially reprogrammed iPS cells and the half way state of blastema cells and suggest that the processes leading to these two cell types might be regulated by some common mechanisms.

## Methods

### Animal maintenance

Zebrafish (AB strain) were maintained in tanks with recirculating water system under standard conditions [59]. Xenopus laevis tadpoles were kept in a recirculating system at 23°C and fed twice a day until the required stage [60].

### Embryo collection and blastema recovery

Zebrafish embryos were washed for two minutes with a 0.5% bleach solution, rinsed twice with embryo medium (EM) and kept in fresh EM at 28°C until they reached the oblong stage (3 2/3 h) [61].

For blastema recovery, fish were anaesthetized in tricain, caudal fins were partially amputated and animals were then kept in system tank until sample recovery. Samples were recovered at different times: 0 hours post-amputation (hpa), 6 hpa (when a thin layer of wound epidermis appears), 24 hpa (wound epidermis), 48 hpa (functional blastema), 3 dpa and 4 dpa. Fin samples from six animals were pooled for each replicate and four to seven replicates have been done.

For *Xenopus *animal caps, embryos were obtained by artificial fertilization and cultured in 0.1 Modified Barth's Saline (MBS) until stage 9 at which point 20 animal caps were excised and pooled for RNA extraction. For tail and limb transections tadpoles were anaesthetized in 1/3000 MS222 in 0.1 × MMR and transferred to a moistened paper towel. For tail amputations, the posterior 50% of the tail was removed with iridectomy scissors. Limbs (st52 or st57) were amputated bilaterally at mid zeugopod level. After amputation tadpoles were let to recover in 0.1 × MMR and kept and fed in the lab in individual tanks till blastema recovery. Blastemas (including about 200 μm of the distal stump tissue) of 20 tadpoles each were recovered at 0 dpa, 1 dpa, 3 dpa and 5 dpa and pooled for RNA extraction. Two to three replicas have been done for each time point.

### RNA extraction and cDNA Synthesis

Total RNA was extracted from zebrafish embryos, cap cells or blastemas using TRIZOL^® ^method according to the manufacturer's guidelines (Invitrogen S.A., Barcelona, Spain) RNA concentration was measured using a NanoDrop spectrophotometer (ND-1000) and 0,2-1 μg total RNA was used for reverse transcription in a 20 μl mixture containing 1 μl of 50 μM Oligo(dT) primer, 2 μl 10 mM dNTP Mix, 4 μl 5× cDNA synthesis Buffer, 1 μl 0,1 MDTT, 1 μl RnaseOUT, 1 μl Cloned AMV RT (15 units/μl) (Invitrogen S.A, Barcelona, Spain) and DEPC-treated water to 20 μl. The reverse transcription was conducted at 50°C for 50 minutes and 85°C for five minutes, and samples were stored at -20°C until use. A pool of 100 embryos was used for each replicate

### Real-Time quantitative PCR

PCR products were detected by measuring the increase in fluorescence caused by the binding of SYBR GREEN dye (Invitrogen, 11760-500) to dsDNA in the reaction tube. For zebrafish 10 μl SYBR were added to 6 μl water, 2 μl sample and 1 μl of each primer (10 μM). For *Xenopus *the cDNA was diluted 1:25 first and then 5 μl of the dilution were combined with 3 μl of water and 1 μl of each primer (10 μM) before 10 μl SYBR green were added. The primers were designed by using the Primer Express (v.3.0) Software from Applied Biosystems Foster City, CA, USA. Primer pairs were chosen to minimize dimerization and to be situated as far as possible towards the 3' end of the mRNA. Relative expression of the PCR products was determined by using the ΔΔCt method [62,63] using as housekeeping gene rpl13A (Zebrafish) or ornithine decarboxylase (ODC) (*Xenopus*) and then normalizing against the highest expression. Each sample was run in duplicate to triplicate and the mean Ct was used in the equation. The primer sets used are shown in Table 1.

### Flow cytometry analysis

For cell cycle analysis, cells were incubated at 37°C for 30 minutes using Hoechst 33342 (H342 - SIGMA, Madrid, Spain) (10 μg/ml final concentration). The tubes were cooled on ice and cells were pelleted by centrifugation (200 g, 5 minutes). The supernatant was removed and cells were resuspended in 0.5 ml of PBS (4°C).

Propidium Iodide (PI - SIGMA, Madrid, Spain) was added at 4 μg/mL (final concentration) to detect dead cells. Immediately after, samples were acquired in a Moflo cell sorter (DakoCytomation (Fort Collins, CO, USA)) adjusted for both UV (351 nm) and blue (488 nm) excitation lines for the detection of H342 (450/65) and PI (670/30) fluorescence respectively. All analyses were performed applying Summit software DakoCytomation (Fort Collins, CO, USA).

### Re-analysis of microarray data sets

Microarray data are available at the NCBI Gene Expression Omnibus database under the following accession numbers: *Xenopus *animal cap - GSE3334 (Dickinson et al., 2006), GSE8990, GSE8496, *Xenopus *regenerating hindlimb - GSE9813 (Pearl et al., 2008), GSE4738 (Grow et al., 2006), zebrafish caudal fin adult - GSE3667 [35], zebrafish caudal fin larval - GSE10184, zebrafish caudal fin adult/larval - GSE10188.

Microarray data was normalized independently for each experiment using GC-RMA in R statistical software http://www.r-project.org. Then for each gene in each sample a relative expression was calculated using a percent rank. It is defined as a rank of the value in a dataset as a percentage of the dataset, and evaluates the relative standing of a value within a dataset. The percent ranks of the genes of interest were obtained from respective probes in each dataset.

The hierarchical clustering was performed using hclust function with the *average *method in R software.

### Immunohistochemistry

For cell division and apoptosis detection, fins were fixed (with PFA 4% for 2 h at 4°C) and used as whole mounts. Fins were first washed three times in Tris buffered saline (TBS) for 10 minutes before they were permeabelised in TBS plus 0.02% Triton and 0.05% tween-20 for 30 minutes at RT. They were than washed twice in TBS for five minutes, incubated in 10 mM Tris-HCl plus 5 mM EDTA at pH 8.5 for 10 minutes before proteinase K treated (20 μg/ml) in the same Tris buffer as before for 15 minutes. Two washes in 5 mM EDTA for five minutes were followed by incubation in the TdT buffer pH7.75 for 10 minutes to equilibrate for the TUNEL reaction. For the TUNEL reaction (2 h at 37°C) the in situ Cell death detection kit TMR red from Roche Diagnostics, Barcelona, Spain was used according to manufacturer's instruction with an enhancement step of the signal. To stop the reaction, fins were washed in Saline-sodium citrate (SSC) plus EDTA buffer twice for 10 minutes, followed by two washes in TBS for 10 minutes. Fins were blocked in TBS plus 6% donkey serum twice for 10 minutes and then incubated in biotinylated anti-rodamin (1:20) and anti-pH3 (1:500) antibody (rat, Sigma) overnight at 4°C. Next day fins were washed extensively in TBS plus serum before incubated in secondary antibody (Strepdavidine-Alexa568, 1:400; and anti-rat IgG-Cy5, 1:100) overnight at 4°C. Finally samples were washed in TBS, exposed to Dapi for five minutes and mounted to be examined as previously described.

### *In situ *hybridization

The following clones were ordered from ImaGene http://www.imagenes-bio.de: sall4: IRBHp990H1244D, c-myc: IRBHp990A0223D and sox2: IRBHp990B0144D. *In situ *hybridization was done as described in [64]. To make in situ probes sall4 pCMV Sport6 was cut with EcoRV and transcribed with polymerase T7 to make antisense probe and cut with BamHI and transcribed with Sp6 polymerase to make sense probe. C-myc antisense probe was done cutting c-myc pCMV-Sport6.ccdb with KpnI and transcribed with T7 while sense probe was cut with XhoI and transcribed with Sp6. Sox2-pCMV-Sport6 was cut with BamHI to make either antisense or sense probe and transcribed with T7 or Sp6, respectively.

### MO microinjection and *in vivo *electroporation

MO microinjection and electroporation was essentially done as in [40] with a few modifications. Fish were anesthetized with tricaine as previously described and one of 4 MO (GeneTools, Inc. Philomath, OR, USA) was injected into the dorsal side of a 0 to 3 dpa fin blastema grown at 28°C. Immediately after the injection of the blastema distal to each dorsal bony ray with MO, the whole fin was electroporated using a CUY21 Square Wave Electroporator and CUY647-5 × 10 tweezer electrodes (Nepa Gene Co, Ldt, Japan). We used the same parameters as Thummel et al.; with 10 consecutive 50 msec pulses at 15V with a one second pause between pulses and an approximately 2 mm gap between the tweezer electrodes. As conducting gel we used 3% methylcellulose. Each fish was tracked individually to calculate the regeneration progress over time. Zebrafish fins were imaged just after MO injection/electroporation and again 24 h later. The area of both dorsal and ventral regrowth was measured using the MetaMorph office programme (Molecular Devices Corporation, USA). Percentage of overall fin regrowth was determined as (D/V)x100 (where D is dorsal blastema and V is ventral blastema 24 h after MO injection). Growth over the last 24 h was calculated by (Dx-D(x-1))×100/(Vx-V(x-1)) (where Dx is dorsal blastema at day x and D(x-1) is dorsal blastema on the day before, Vx is ventral blastema at day x and V(x-1) is ventral blastema on day before, making sure that D(x-1) and V(x-1) (dorsal and ventral blastema on day of morpholino injection) are of equal size. N = 5 for dextran 2d, n > 20 for pou MO 3d, n = 8 to 12 for rest.

Morpholinos used are: msxb MO; TTAACCATCCGCCACGAGCTGCTGC, pou MO; CGCTCTCTCCGTCATCTTTCCGCTA, pou 5-mis MO, CGGTCTGTCCGTGATCTTTGCGGTA, sox2 MO; GCTCGGTTTCCATCATGTTATACA. Each morpholino contained a 3'fluorescein tag and was resuspended as a 2 mM solution in water and either injected at 1.2 mM (msxb MO) or 2 mM (sox2 MO, pou MO, pou 5-mis MO).

### Statistical analysis

In zebrafish, qPCR data were analyzed with SPSS software (SPSS Inc., Chicago, IL, USA) (SPSS Statistics 17.0) using a repeated measures linear mixed-effect model (LME) to test the significance of changes in gene expression. Oblong stage (for pluripotency associated markers) and 48 hpa (for blastema markers) were used as intercept for the models, whereas replicates were used as a grouping factor (random effect). Results in Additional file 1 show the expression effect size at each time point (mean+-SEM) compared to oblong stage and 48 hpa, respectively. For verifying *Xenopus *qPCR or the morpholino knockdown results a student t-test was performed.

### Animal Welfare

All animal experiments were done with the approval of the institutions ethical committee.

## Abbreviations

dpa: days post amputation; ES: embryonic stem; FACS: fluorescence-activated cell sorting; G0: gap phase 0; G1: gap phase 1; G2: gap phase 2; hpa: hours post amputation; hpf: hours post fertilisation; iPS: induced pluripotent stem; M: Mitosis; MMR: Marc's modified Ringers; MO: morpholino; pH3: phospho-Histone H3; qPCR: quantitative real time polymerase chain reaction; st: stage; TBS: Tris buffered saline; TUNEL: terminal deoxynucleotidyl transferase-mediated dUTP nick end labelling.

## Authors' contributions

BC participated in design of the study, collected all the *Xenopus *data, participated in the collection and interpretation of morpholino knockdown and drafted the manuscript. VR participated in design of the study, collected and interpreted all zebrafish qPCR data, collection and interpretation of FACS results, participated in setting up and interpretation of morpholino knockdown and drafted the manuscript. MR helped with design of study, participated in morpholino knockdown study and helped with manuscript. IP provided data analysis and interpretation of microarray reanalysis and helped with manuscript. JCIB participated in design of the study, financial support and helped with manuscript writing.

## Supplementary Material

Additional file 1 **Figure S1: Statistical relevance of changes in expression levels**. Effect sizes (mean ± error) for each regeneration time point on the intercept, for zebrafish pluripotency associated makers and blastema markers. Oblong stage (for pluripotency markers) and 48 hpa (for blastema markers) were the intercept of the models. Asterisks represent statistical differences with the intercept. Click here for file

Additional file 2 **Figure S2: Cell divisions in non- regenerating and regenerating fin**. Immunohistochemistry of phospho-Histone H3 localization in zebrafish fins: 0 hours (A) and 48 hours (B) post amputation. Click here for file
