# Supplementary figures and images for: Regeneration and reprogramming compared
Source: BMC Biol. 2010 Jan 20;8:5. doi: 10.1186/1741-7007-8-5 (PMC2826312; doi:10.1186/1741-7007-8-5)

Figure 1S

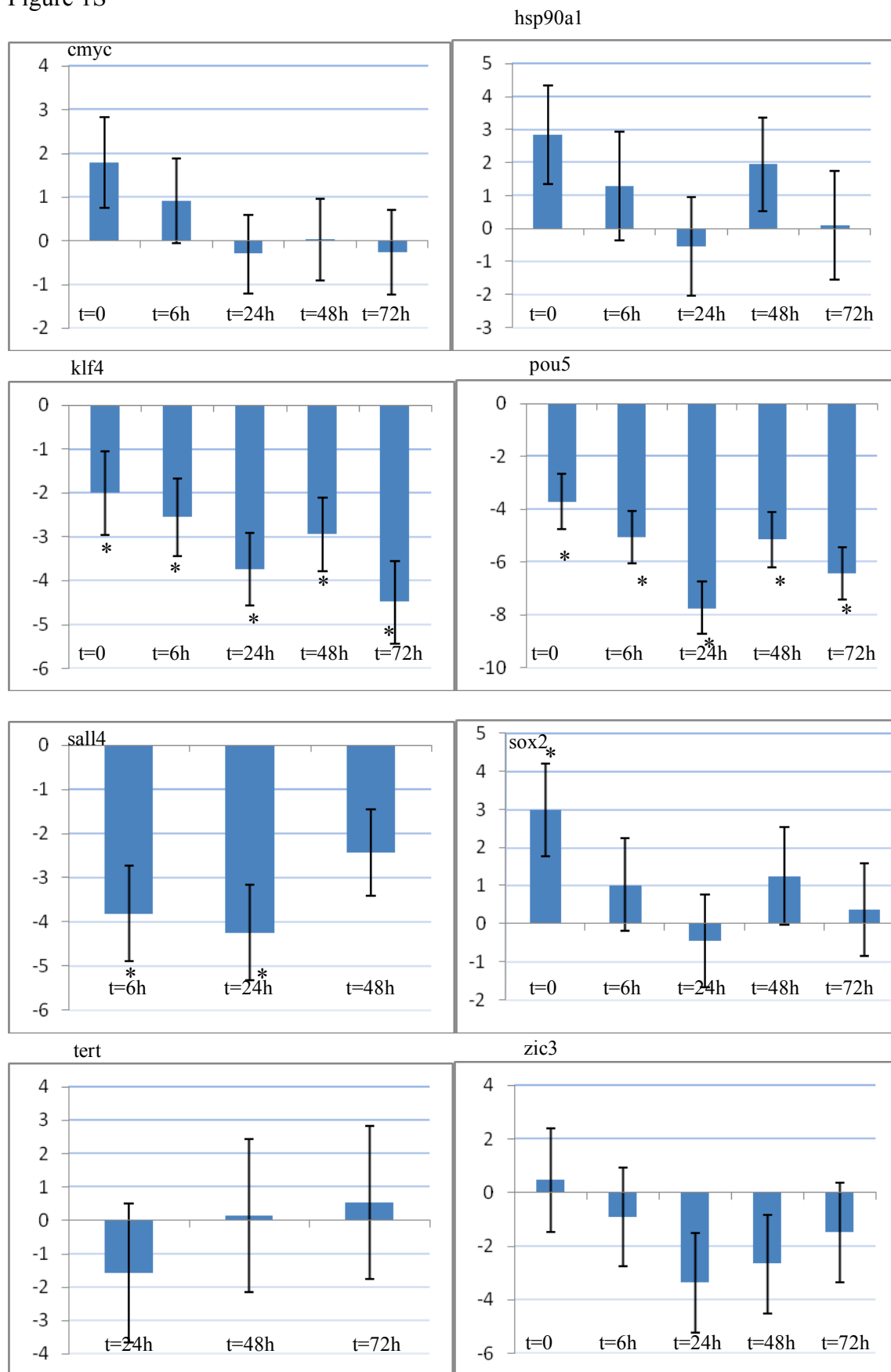

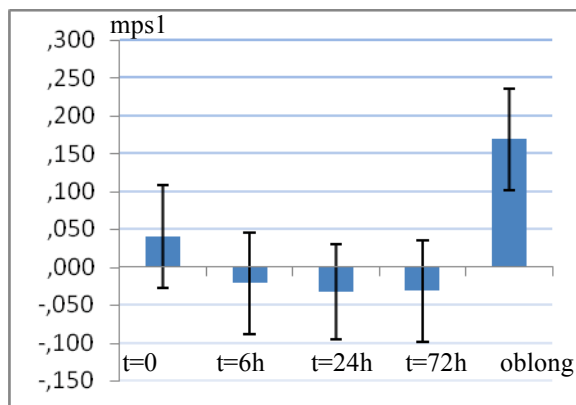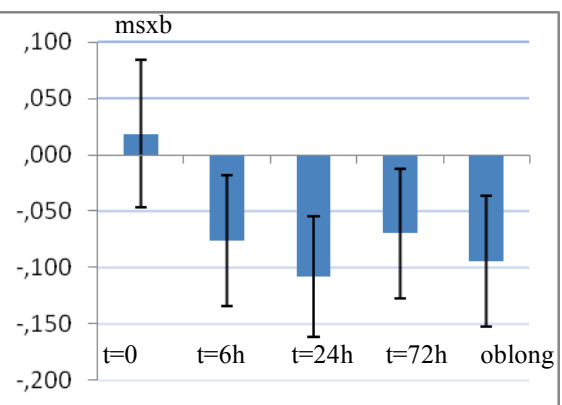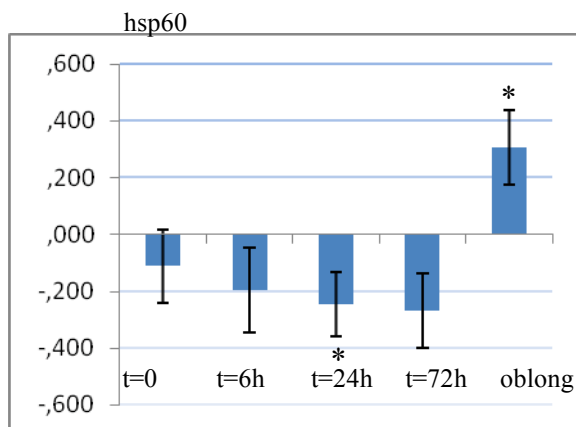

Supplement: Additional file 1 — Figure S1: Statistical relevance of changes in expression levels. Effect sizes (mean ± error) for each regeneration time point on the intercept, for zebrafish pluripotency associated makers and blastema markers. Oblong stage (for pluripotency markers) and 48 hpa (for blastema markers) were the intercept of the models. Asterisks represent statistical differences with the intercept. [file 1741-7007-8-5-S1.PDF]

Figure 2S

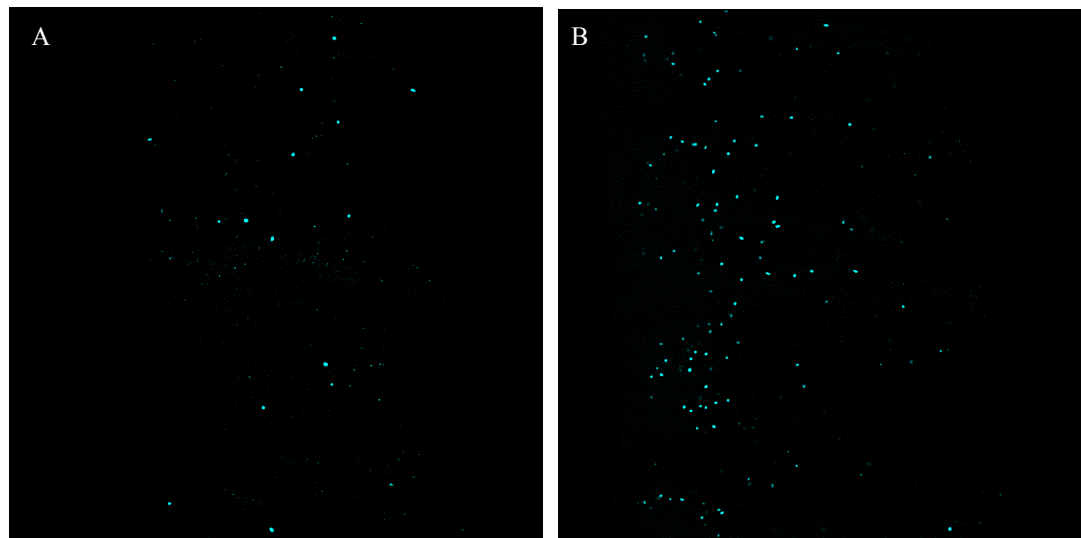

Supplement: Additional file 2 — Figure S2: Cell divisions in non- regenerating and regenerating fin. Immunohistochemistry of phospho-Histone H3 localization in zebrafish fins: 0 hours (A) and 48 hours (B) post amputation. [file 1741-7007-8-5-S2.PDF]
